# Estonian hip fracture data from 2009 to 2017: high rates of non­operative management and high 1-year mortality

**DOI:** 10.1080/17453674.2018.1562816

**Published:** 2019-01-23

**Authors:** Pärt Prommik, Helgi Kolk, Pirja Sarap, Egon Puuorg, Eva Harak, Andres Kukner, Mati Pääsuke, Aare Märtson

**Affiliations:** a University of Tartu;; b Tartu University Hospital, Estonia

## Abstract

Background and purpose — There are no national guidelines for treatment of hip fractures in Estonia and no studies on management. We assessed treatment methods and mortality rates for hip fracture patients in Estonia.

Patients and methods — We studied a population-based retrospective cohort using validated data from the Estonian Health Insurance Fund’s database. The cohort included patients aged 50 and over with an index hip fracture diagnosis between January 1, 2009 and September 30, 2017. The study generated descriptive statistics of hip fracture management methods and calculated in-hospital, 1-, 3, 6-, and 12-month unadjusted all-cause mortality rates. [CrossRef]

Results — 91% (number of hips: 11,628/12,731) of the original data were included after data validation. Median patient age was 81 years, 83 years for women and 74 years for men. 28% were men. Treatment methods were: total hip arthroplasty 7%; hemiarthroplasty 25%; screws 6%; sliding hip screw 25%; intramedullary nail 27%; and nonoperative management 10%. Unadjusted all-cause mortality rates for in-hospital, 1, 3, 6, and 12 months were: 3%, 9%, 18%, 24%, and 31% respectively. The 12-month mortality rate for nonoperative management was 58%. [CrossRef]

Interpretation — High rates of nonoperative management and overall high 1-year mortality rates after an index hip fracture indicate the need to review exclusion criteria for surgery and subacute care in Estonia.

Baseline characteristics of patients presented as total, operative (OM), and nonoperative management (NOM) and statistical difference between the last two. Values are n (%) unless otherwise specified

There has been no change in mortality rates for hip fracture (HF) over the last 3 decades, regardless of advancements in surgical solutions and regardless of the fact that the majority of patients now are operated on (Mundi et al. 2014, Johansen et al. 2017). With the total number of HFs predicted to increase, the associated socioeconomic burden will become an even more challenging problem in the future (Gullberg et al. 1997, Cheung et al. 2018).

The most appropriate healthcare strategies to address this challenging problem will be those based on the valid conclusions of high-quality research, which requires accurate data. Quality of administrative data can be improved with validation and this may lead to more accurate conclusions, which are needed for effective treatment guidelines. Currently there are no national guidelines for HF in Estonia and its management is unstudied. The management-specific outcomes may contribute towards the development of healthcare strategies and clinical practice.

Therefore we assessed the relative prevalence of HF management methods in Estonia and calculated the mortality rates.

## Patients and methods

We conducted a population-based retrospective cohort study based on validated medical bill data from the Estonian Health Insurance Fund (EHIF), which insures 94% of the Estonian population. The data of HF patients in Estonia without EHIF insurance was also included. The cohort included those patients aged 50 and over with an index HF diagnosis between January 1, 2009 and September 30, 2017. HF diagnosis was based on the International Classification of Diseases (ICD-10) codes: S72.0—fracture of femoral neck; S72.1—pertrochanteric fracture; and S72.2—subtrochanteric fracture.

The following data were extracted for analysis: anonymized patient identification number (ID); age at hospitalization; sex; admission date; discharge date; fracture type; death date; date(s) of operation(s) within a year of hospitalization; and the Nordic Medico-Statistical Committee’s Classification of Surgical Procedures code (NCSP). Patients’ comorbidities (ICD-10 codes) were queried 4 years prior to index HF to calculate the Charlson comorbidity index (CCI) using updated weights and coding algorithms according to Quan et al. (2005, 2011). Only codes that appeared at least twice and at least 7 days apart were included to increase the method’s validity. Data on any HF diagnoses prior to the study period were also extracted. For patients without operative management data, personal identification codes and healthcare service codes used for orthopedic operations’ funding were extracted. Patients’ operative and mortality statuses were finalized as at December 31, 2017 and October 10, 2018, respectively.

We used a multi-step strategy to validate the data. First, patients with a known prior HF were excluded. Second, a logic check was used and patients with both an HF diagnosis and an appropriate NCSP code within 3 months of diagnosis were included. The appropriate NCSP codes for operative management (OM) methods were: total hip arthroplasty, THA (NFB20, NFB30, NFB40, NFB99); hemiarthroplasty, HA (NFB00-9; NFB10-9); screws (NFJ70-3); sliding hip screw, SHS (NFJ60-3, NFJ80-3); intramedullary nail, IMN (NFJ50-3). Finally, the digital imaging and reports of those patients without operation information were reviewed, as well as the controlled operation codes used for funding. If this review did not show that a patient met the inclusion criteria, then the patient’s medical records were examined. For validation purposes, 2 national databases were used: Foundation of Estonian PACS (an image archiving and communication system database) and Electronic Health Record (e-Health Record). Digital imaging was reviewed by a radiologist (PS) and an orthopedic surgeon (EP). Fractures were initially classified by EHIF database and confirmed by an agreement as follows: the radiologist and report; the orthopedic surgeon and report; radiologist and orthopedic surgeon (no report). Medical records were reviewed by an orthogeriatrician (HK).

For comparisons of sex proportions in age subgroups with the general population, the Statistics Estonia online database with summary-level data was used (http://pub.stat.ee/). However, only years 2016 and 2017 were included, since only these contained sufficiently detailed information for age subgroups.

### Statistics

For continuous variables with normal distribution, mean and standard deviations (SD) are shown. For continuous variables with non-normal distribution, median (range) is shown. Categorical variables are shown as proportions. Multiple analyses were performed on the following variables: age, divided into 10-year subgroups; fracture type, grouped as intracapsular (S72.0) and extracapsular (S72.1 and S72.2); management method, grouped as nonoperative management (NOM) and OM; OM, grouped as THA, HA, screws, SHS, IMN; and temporal change, divided into two 4-year periods (2009–2012 and 2013–2016). In-hospital mortality was calculated only for stationary acute care patients. Patients hospitalized in 2017 were excluded from any analyses that required full-year data.

Age and CCI were non-normally distributed by the Kolmogorov-Smirnov test (p < 0.001 for both) and were therefore analyzed using a Mann–Whitney U-test (Wilcoxon rank-sum test). Sex proportions within age subgroups were compared with those of the general population using a binominal test. The Mantel–Haenszel test for trend (Linear-by-Linear Association test in IBM SPSS Statistics software; IBM Corp, Armonk, NY, USA) was used to find linear associations. The Pearson chi-square test was used for proportional comparisons. Kaplan–Meier unadjusted cumulative all-cause mortality analyses were conducted at the end of hospitalization and at 1, 3, 6, and 12 months afterwards. The log-rank test was used to compare cumulative mortality between groups. Cox proportional hazard regression analysis was used to estimate age, sex, and CCI adjusted differences in mortality risk between groups. Hazard ratios are presented with 95% confidence intervals (CI). Statistical significance was defined as p < 0.05 and all tests were 2-sided. All statistical analyses were conducted using statistical software IBM SPSS Statistics for Windows, version 25 (IBM Corp, Armonk, NY, USA).

### Ethics, registration, funding, and potential conflicts of interest

The study was approved by the Research Ethics Committee of the University of Tartu on June 17, 2013 (reference 227/T-12). Additional approval from the Estonian Data Protection Inspectorate for the use of personalized data was received on December 1, 2017 (reference 2.2.-1/17/47). This work was supported by the following projects: Interreg Baltic Sea Region Programme 2014-2020 (grant number: #R001); Estonian Science Agency project IUT20-46 (TARBS14046I); HypOrth Project funded by the European Union’s 7th Framework Programme grant agreement no. 602398; Institutional Research Funding IUT20-58 of the Estonian Ministry of Education and Research. No conflicts of interest were declared.

## Results

### Data validation

**Figure 1.**
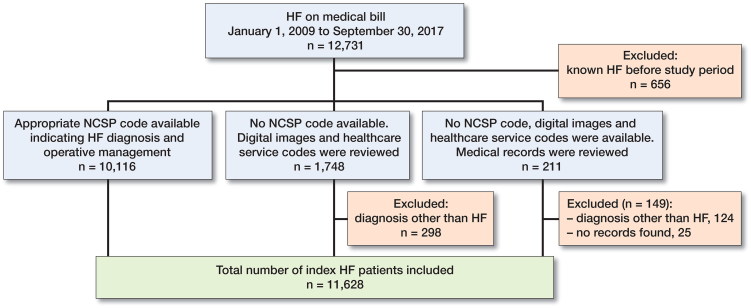
Data validation process. HF = hip fracture; NCSP = the Nordic Medico-Statistical Committee’s Classification of Surgical Procedures code.

After data validation 91% (11,628/12,731) of the original population data were included (Figure 1). Almost all patients (99%; 11,500/11,628), had health insurance. In the period between 2009 and 2016, a mean of 1,328 (SD 66) patients per year received an HF diagnosis.

### Patients and management

**Figure 2.**
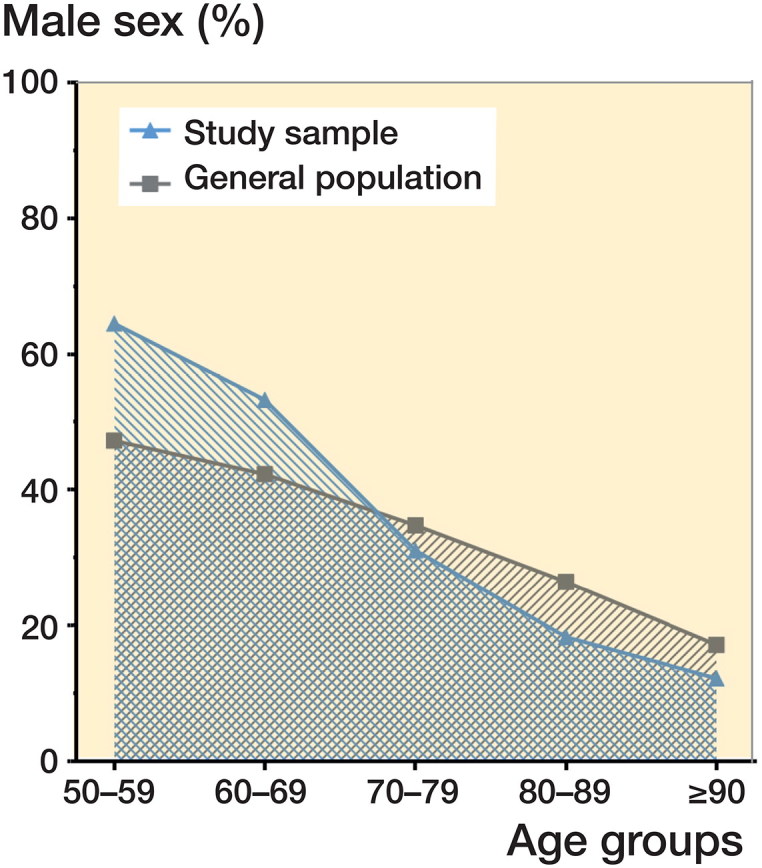
Relative proportion of men by age as compared with the general population. *p < 0.001 difference between populations’ age subgroups.

Median patient age was 81 years (50–104). The proportion of men was 28% (3,287/11,628) and they were 9 years younger (Table). Men were in the majority in the 2 youngest age subgroups. There was a statistically significant linear trend for the proportion of men to decrease as age increased; the reverse was found for women (p < 0.001). The proportion of men in each age subgroup differed from that of the general population as follows: 17% higher for 50–59 years; 11% higher for 60–69 years; 3.8% lower for 70–79 years; 8.1% lower for 80–89 years; and 4.9% lower for 90 years and over (p < 0.001 for all) (Figure 2).

**Figure 3.**
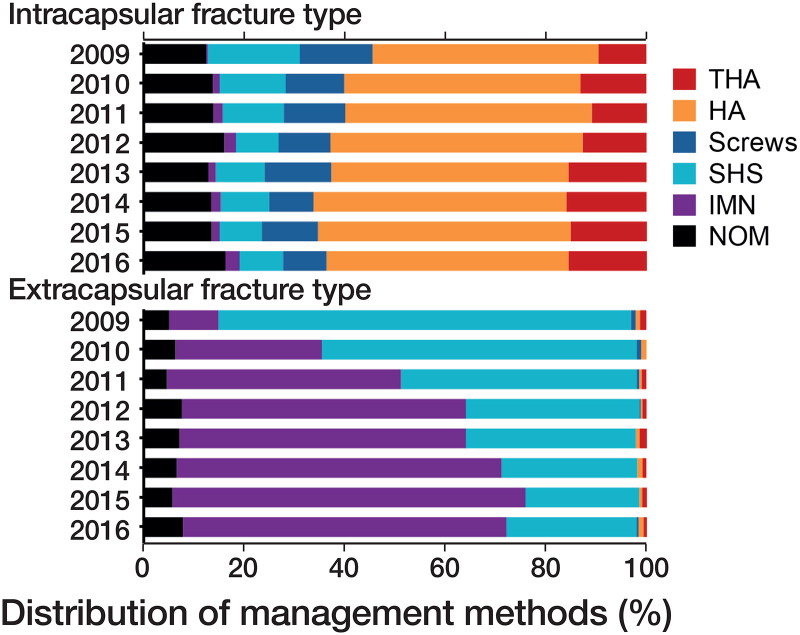
Distribution of management methods by study year and fracture type. THA = total hip arthroplasty, HA = hemiarthroplasty, SHS = sliding hip screw, IMN = intramedullary nail, NOM = nonoperative management.

90% (10,431/11,628) of patients received operative management. Operative methods were: THA 7.4% (861/11,628); HA 25% (2,949/11,628); screws 5.8% (679/11,628); SHS 25% (2,856/11,628); IMN 27% (3,086/11,628). The operation date was available for 99% (10,372/10,431) of operated patients: 72% (7,461/10,431) were operated on within the first 2 days of hospitalization. Temporal changes in management methods of note (> 1%) between 2009–2012 and 2013–2016 for intracapsular fractures were: 3.9% increase for THA; 1.1% increase for HA; 1.7% decrease for screws; 3.9% decrease for SHS (p < 0.001). The same estimates for extracapsular fractures were: 29% decrease for SHS; 28% increase for IMN (p < 0.001) (Figure 3). In comparison with OM, NOM patients had higher median age; a higher proportion of patients aged 50–59 or ≥ 90 years; a higher proportion of femoral neck fracture; more comorbidities (Table).

### Mortality

**Figure 4.**
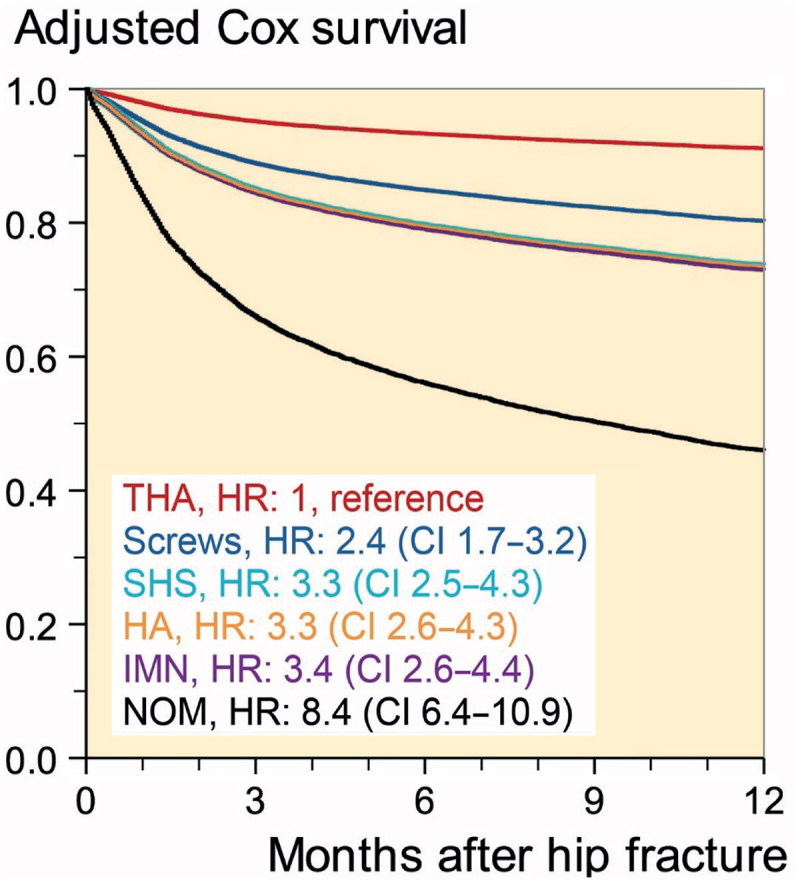
Cox survival curves adjusted for age, sex, and Charlson comorbidity index score. For abbreviations, see Figure 3 caption.

In-hospital, 1-, 3-, 6-, and 12-month unadjusted all-cause mortality rates were: 3.2%; 8.6%; 18%; 24%; and 31%, respectively. Unadjusted all-cause cumulative 1-year mortality rates for women and men were similar (31%). However, age and comorbidity adjusted analysis showed the 1-year mortality risk for men to be 1.6 times higher (HR 1.6 [CI 1.5–1.7]). Overall mortality rate for the combined operative management methods was 28%, as compared with the NOM rate of 58% (p < 0.001). When adjusted for age, sex, and CCI, the 1-year mortality risk for NOM was 2.6 times higher than for operative management (HR 2.6 [CI 2.4–.9]) (Figure 4).

## Discussion

Distribution of operative management methods and time trends were similar to those of other studies (Gjertsen et al. 2017, Johansen et al. 2017). However, the NOM rate was unexpectedly high, being 1.6–10 times higher than NOM rates reported in other general population studies (Neuman et al. 2010 [USA], Cram et al. 2017 [Canada], Johansen et al. 2017 [Sweden, England, Wales, Northern Ireland, Scotland, Ireland, New Zealand, Australia]). The rate is comparable to that reported for nursing home residents (Berry et al. 2009, Neuman et al. 2014). Furthermore, the 12-month mortality rates for NOM patients were 10-17% higher than those of comparable studies (Cram et al. 2017, Ree et al. 2017).

Multiple factors are generally associated with NOM: older age; male sex; more comorbidities; residence in a rural area; femoral neck fractures; residence at baseline long-term care; lower income; and black race (Neuman et al. 2010, Cram et al. 2017). Our findings were consistent with these factors: NOM patients were older; had more comorbidities; and included a higher proportion of men and femoral neck fractures. The study data did not provide information on race, residence, or income. The relatively high NOM rate in Estonia may also be attributable to country-specific factors: traditions and expectations of patients and family; absence of national guidelines; and differences in short- and long-term healthcare support for OM and NOM patients.

Mortality rates for in-hospital and at 1 month were consistent with those of earlier studies (Medin et al. 2015, Johansen et al. 2017). However, mortality rates at 3, 6, and 12 months were higher. For example, the Estonian 3-month mortality rate is comparable to the highest reported rates of a systematic review of 63 studies (Abrahamsen et al. 2009). Multiple studies, including a systematic review, have reported lower mortality rates at 1 year than the Estonian rate at 6 months (Kurtinaitis et al. 2012, Diamantopoulos et al. 2013, Brozek et al. 2014, Klop et al. 2014, Mundi et al. 2014, Poenaru et al. 2014). However, relatively similar 12-month mortality rates have been reported in Denmark, Hungary, and Scotland (Medin et al. 2015, Jantzen et al. 2018). Delayed surgery may also contribute to the high mortality rates; however, our result is similar to the findings of a recent study (Johansen et al. 2017).

Relatively high mortality rates from the 3rd month onwards may be attributable in part to high rates of NOM that showed a higher 1-year mortality risk compared with every operative management type. Also, the crude 1-year mortality rate for operatively managed patients was lower than that of the overall study population. Differences in mortality from the third month onwards may be related to shortcomings in subacute care, such as accessibility of rehabilitation. This is supported by a study that compared the Estonian HF group with a non-fracture reference group using age and comorbidity adjusted relative risk ratios. The relative risk ratios are higher, especially for HF women at 3 and 12 months in Estonia, compared with the findings of a systematic review (Haentjens et al. 2010, Jürisson et al. 2017). The same estimates for men are near the upper confidence limits reported in the review article. On the other hand, previous studies have shown various preoperative indicators to be associated with increased HF mortality risk: advanced age; male sex; pre-fracture functional status; residence in an institutional care home; presence of an intra-capsular fracture; cognitive impairment; depression; and more comorbidities (Hu et al. 2012, Liu et al. 2017).

We found similar unadjusted mortality rates for men and women. However, the median age of men was lower, and age-adjusted analyses showed a higher mortality risk for men. These results are consistent with those of other studies (Abrahamsen et al. 2009, Kannegaard et al. 2010).

The median age of men at the time of HF was relatively low. Other studies have reported a similar median age for female HF, but with a smaller age difference between the sexes (Kannegaard et al. 2010, Kurtinaitis et al. 2012, Diamantopoulos et al. 2013, Klop et al. 2014). The Estonian HF population has 5% more patients below the age of 80 years compared with an Austrian study by Brozek et al. (2014). There also was a difference in the proportion of sexes in age subgroups. In Estonia, men were highly prevalent in each of the 2 youngest age subgroups, whereas in Austria 10% fewer men were in the 50–59 and 60–69 age subgroups and more men were older (Brozek et al. 2014). The prevalence of men was also higher in the 2 youngest age subgroups, as compared with the general population. Jürisson et al. (2015) proposed that the incidence of Estonian male HF may be explained by relatively high rates of alcohol consumption, with a consequently greater risk of alcohol-related falls and injuries.

The problem of flawed data in databases has been reported in previous studies on HF populations (Cundall-Curry et al. 2016). They suggested that data should not be used from administrative databases without validation, because conclusions based on inaccurate data may be erroneous and may misinform clinical practice and policy development. Our study followed a novel strategy of multi-step data validation to improve the quality of data extracted from a large administrative database. This strategy enabled a relatively high proportion of unsuitable cases (8.7%) to be excluded from analysis.

Our study has multiple strengths thT increase the generalizability of the results: validated high-quality, whole-population data; unbiased and standardized data collection; and up-to-date data, with a long (9-year) study period. However, some limitations must be acknowledged. First, the data validation process may not have yielded the same level of accuracy as would the review of individual patient data case-by-case; however, individualized review is time-consuming and would involve processing an unnecessary amount of personalized data. In contrast, our study’s logical data validation process enabled the reduction of personal data use to just 14%. Second, the EHIF database does not contain information on patients who pay for their own care. The number of these patients in Estonia would be negligible, however, since emergency medical care is guaranteed and all Estonian citizens have health insurance on retirement. Third, the EHIF database dates back only to 2004. Study data may therefore have included patients with secondary HF. Secondary HF is associated with increased risk of death and may therefore have affected the results (Sobolev et al. 2015). Finally, the study data did not provide information on patients’ residence and lifestyle factors, which could have informed some of the issues raised in the discussion.

In summary, this is the first study to report management-specific outcomes for HF in Estonia. The study identified several issues that merit further attention in clinical practice and research. Clinical practice should be reviewed with an aim to lower NOM and the 1-year mortality rate. Further research is already underway on the NOM decision-making process and on the long-term use of rehabilitation after HF.

Contributions of the authors were as follows: PP, HK, MP, and AM designed the study; PP, PS, EP, and HK validated data; PP and EH performed data analysis and wrote the first draft; HK, AK, MP, and AM jointly revised the manuscript to its final form.

**Table:** 

	Total n = 11,628	OM n = 10,431	NOM n = 1,197	p-value
Men	3,287 (28)	2,912 (28)	375 (31)	0.01
Median age (range)	81 (50–104)	81 (50–102)	82 (50–104)	< 0.001
Age subgroups:				< 0.001
50–59	804 (6.9)	720 (6.9)	84 (7.0)	
60–69	1,422 (12)	1,298 (12)	124 (10)	
70–79	2,984 (26)	2,702 (26)	282 (24)	
80–89	4,913 (42)	4,442 (43)	471 (39)	
≥ 90	1,505 (13)	1,269 (12)	236 (20)	
Fracture type:				< 0.001
Femoral neck	5,988 (52)	5,145 (49)	843 (70)	
Pertrochanteric	4,967 (43)	4,663 (45)	304 (25)	
Subtrochanteric	673 (5.8)	623 (6.0)	50 (4.2)	
CCI, mean (SD)	1.7 (1.7)	1.6 (1.6)	2.0 (1.8)	< 0.001
Comorbidities:				
Myocardial infarction	809 (7.0)	725 (7.0)	84 (7.0)	0.9
Congestive heart failure	5,097 (44)	4,520 (43)	577 (48)	0.001
Peripheral vascular disease	1,219 (10)	1,060 (10)	159 (13)	0.001
Cerebrovascular disease	2,504 (22)	2,225 (21)	279 (23)	0.1
Dementia	1,121 (9.6)	913 (8.8)	208 (17)	< 0.001
Chronic pulmonary disease	1,259 (11)	1,117 (11)	142 (12)	0.2
Rheumatic disease	388 (3.3)	349 (3.3)	39 (3.3)	0.9
Peptic ulcer disease	550 (4.7)	488 (4.7)	62 (5.2)	0.4
Mild liver disease	175 (1.5)	154 (1.5)	21 (1.8)	0.4
Diabetes				
without chronic complication	1,264 (11)	1,132 (11)	132 (11)	0.8
with chronic complication	688 (5.9)	619 (5.9)	69 (5.8)	0.8
Hemi- or paraplegia	536 (4.6)	475 (4.6)	61 (5.1)	0.4
Renal disease moderate/severe	473 (4.1)	414 (4.0)	59 (4.9)	0.1
Any malignancy	1,193 (10)	1,042 (10)	151 (13)	0.005
Moderate/severe liver disease	36 (0.31)	27 (0.26)	9 (0.75)	0.004
Metastatic solid tumor	42 (0.36)	38 (0.36)	4 (0.33)	0.9
AIDS/HIV	1 (0.01)	1 (0.01)	0 (0)	0.7

CCI = Charlson Comorbidity Index

## References

[CIT0001] Abrahamsen B, Staa T van, Ariely R, Olson M, Cooper C Excess mortality following hip fracture: a systematic epidemiological review. Osteoporos Int 2009; 20(10): 1633–50. 19421703 10.1007/s00198-009-0920-3

[CIT0002] Berry S D, Samelson E J, Bordes M, Broe K, Kiel D P Survival of aged nursing home residents with hip fracture. J Gerontol A Biol Sci Med Sci 2009; 64A(7): 771-7. 10.1093/gerona/glp019 PMC2844133 19414511

[CIT0003] Brozek W, Reichardt B, Kimberger O, Zwerina J, Dimai H P, Kritsch D, Klaushofer K, Zwettler E Mortality after hip fracture in Austria 2008–2011. Calcif Tissue Int 2014; 95(3): 257–66. 24989776 10.1007/s00223-014-9889-9

[CIT0004] Cheung C-L, Ang S B, Chadha M, Chow E S-L, Chung Y-S, Hew F L, Jaisamrarn U, Ng H, Takeuchi Y, Wu C-H, Xia W, Yu J, Fujiwara S An updated hip fracture projection in Asia: the Asian Federation of Osteoporosis Societies study. Osteoporos Sarcopenia 2018; 4(1): 16–21. 10.1016/j.afos.2018.03.003 PMC6362950 30775536

[CIT0005] Cram P, Yan L, Bohm E, Kuzyk P, Lix L M, Morin S N, Majumdar S R, Leslie W D Trends in operative and nonoperative hip fracture management 1990-2014: a longitudinal analysis of Manitoba administrative data. J Am Geriatr Soc 2017; 65(1): 27–34. 27861712 10.1111/jgs.14538

[CIT0006] Cundall-Curry D J, Lawrence J E, Fountain D M, Gooding C R Data errors in the National Hip Fracture Database. Bone Joint J 2016; 98-B(10): 1406–9. 27694597 10.1302/0301-620X.98B10.37089

[CIT0007] Diamantopoulos A P, Hoff M, Skoie I M, Hochberg M, Haugeberg G Short- and long-term mortality in males and females with fragility hip fracture in Norway: a population-based study. Clin Interv Aging 2013; 8: 817–23. 23861581 10.2147/CIA.S45468 PMC3704300

[CIT0008] Gjertsen J-E, Dybvik E, Furnes O, Fevang J M, Havelin L I, Matre K, Engesaeter L B Improved outcome after hip fracture surgery in Norway. Acta Orthop 2017; 88(5): 505–11. 28681677 10.1080/17453674.2017.1344456 PMC5560213

[CIT0009] Gullberg B, Johnell O, Kanis J A World-wide projections for hip fracture. Osteoporos Int J Establ Result Coop Eur Found Osteoporos Natl Osteoporos Found USA 1997; 7(5): 407–13. 10.1007/pl00004148 9425497

[CIT0010] Haentjens P, Magaziner J, Colon-Emeric C, Vanderschueren D, Milisen K, Velkeniers B, Boonen S Meta-analysis: excess mortality after hip fracture among older women and men. Ann Intern Med 2010; 152(6): 380–90. 20231569 10.1059/0003-4819-152-6-201003160-00008 PMC3010729

[CIT0011] Hu F, Jiang C, Shen J, Tang P, Wang Y Preoperative predictors for mortality following hip fracture surgery: a systematic review and meta-analysis. Injury 2012; 43(6): 676–85. 21683355 10.1016/j.injury.2011.05.017

[CIT0012] Jantzen C, Madsen C M, Lauritzen J B, Jørgensen H L Temporal trends in hip fracture incidence, mortality, and morbidity in Denmark from 1999 to 2012. Acta Orthop 2018; 89(2): 170–6. 29388458 10.1080/17453674.2018.1428436 PMC5901514

[CIT0013] Johansen A, Golding D, Brent L, Close J, Gjertsen J-E, Holt G, Hommel A, Pedersen A B, Röck N D, Thorngren K-G Using national hip fracture registries and audit databases to develop an international perspective. Injury 2017; 2174–9. 28803651 10.1016/j.injury.2017.08.001

[CIT0014] Jürisson M, Vorobjov S, Kallikorm R, Lember M, Uusküla A The incidence of hip fractures in Estonia, 2005–2012. Osteoporos Int 2015; 26(1): 77–84. 25182229 10.1007/s00198-014-2820-4

[CIT0015] Jürisson M, Raag M, Kallikorm R, Lember M, Uusküla A The impact of hip fracture on mortality in Estonia: a retrospective population-based cohort study. BMC Musculoskelet Disord 2017; 18(1): 243–52. 28583096 10.1186/s12891-017-1606-1 PMC5460499

[CIT0016] Kannegaard P N, Mark S van der, Eiken P, Abrahamsen B Excess mortality in men compared with women following a hip fracture: national analysis of comedications, comorbidity and survival. Age Ageing 2010; 39(2): 203–9. 20075035 10.1093/ageing/afp221

[CIT0017] Klop C, Welsing P, Cooper C, Harvey N, Elders P, Bijlsma J, Leufkens H, de Vries F Mortality in British hip fracture patients, 2000–2010: a population-based retrospective cohort study. Bone 2014; 66: 171–7. 24933345 10.1016/j.bone.2014.06.011

[CIT0018] Kurtinaitis J, Dadonienė J, Kvederas G, Porvaneckas N, Butėnas T Mortality after femoral neck fractures: a two-year follow-up. Med Kaunas Lith 2012; 48(3): 145–9. 22588346

[CIT0019] Liu Y, Wang Z, Xiao W Risk factors for mortality in elderly patients with hip fractures: a meta-analysis of 18 studies. Aging Clin Exp Res 2017; 30(4): 323–30. 10.1007/s40520-017-0789-5 28660596

[CIT0020] Medin E, Goude F, Melberg H O, Tediosi F, Belicza E, Peltola M, on behalf of the EuroHOPE study group European regional differences in all-cause mortality and length of stay for patients with hip fracture. Health Econ 2015; 24: 53–64. 26633868 10.1002/hec.3278

[CIT0021] Mundi S, Pindiprolu B, Simunovic N, Bhandari M Similar mortality rates in hip fracture patients over the past 31 years. Acta Orthop 2014; 85(1): 54–9. 24397744 10.3109/17453674.2013.878831 PMC3940992

[CIT0022] Neuman M D, Fleisher L A, Even-Shoshan O, Mi L, Silber J H Non-operative care for hip fracture in the elderly: the influence of race, income, and comorbidities. Med Care 2010; 48(4): 314–20. 20355262 10.1097/mlr.0b013e3181ca4126 PMC4882126

[CIT0023] Neuman M D, Silber J H, Magaziner J S, Passarella M A, Mehta S, Werner R M Survival and functional outcomes after hip fracture among nursing home residents. JAMA Intern Med 2014; 174(8): 1273–80. 25055155 10.1001/jamainternmed.2014.2362 PMC4122620

[CIT0024] Poenaru D V, Prejbeanu R, Iulian P, Haragus H, Popovici E, Golet I, Vermesan D Epidemiology of osteoporotic hip fractures in Western Romania. Int Orthop 2014; 38(11): 2329–34. 25069426 10.1007/s00264-014-2407-x

[CIT0025] Quan H, Sundararajan V, Halfon P, Fong A, Burnand B, Luthi J-C, Saunders L D, Beck C A, Feasby T E, Ghali W A Coding algorithms for defining comorbidities in ICD-9-CM and ICD-10 administrative data. Med Care 2005; 43(11): 1130–9. 16224307 10.1097/01.mlr.0000182534.19832.83

[CIT0026] Quan H, Li B, Couris C M, Fushimi K, Graham P, Hider P, Januel J-M, Sundararajan V Updating and validating the Charlson comorbidity index and score for risk adjustment in hospital discharge abstracts using data from 6 countries. Am J Epidemiol 2011; 173(6): 676–82. 21330339 10.1093/aje/kwq433

[CIT0027] Ree C L P van de, Jongh M A C D, Peeters C M M, Munter L de, Roukema J A, Gosens T Hip fractures in elderly people: surgery or no surgery? A systematic review and meta-analysis. Geriatr Orthop Surg Rehabil 2017; 8(3): 173–80. 28835875 10.1177/2151458517713821 PMC5557195

[CIT0028] Sobolev B, Sheehan K J, Kuramoto L, Guy P Excess mortality associated with second hip fracture. Osteoporosis Int 2015; 26(7): 1903–10. 10.1007/s00198-015-3104-3 25910745

